# A rare case of atrial flutter and a cystic mass in the left atrium

**DOI:** 10.1136/heartjnl-2024-324714

**Published:** 2024-09-25

**Authors:** Huan Cen, Sinan Chen, Pengtao Sun

**Affiliations:** 1 Ultrasonography, The Second Affiliated Hospital of Guangzhou University of Chinese Medicine, Guangzhou, Guangdong, China

**Keywords:** Echocardiography, Computed Tomography Angiography, Coronary Angiography, Diagnostic Imaging, Cardiovascular Diseases

## Clinical introduction

 A woman is her 60s with no medical history presented to the hospital with palpitations and occasional nausea. The patient reported no chest pain or shortness of breath.

**Figure 1:**
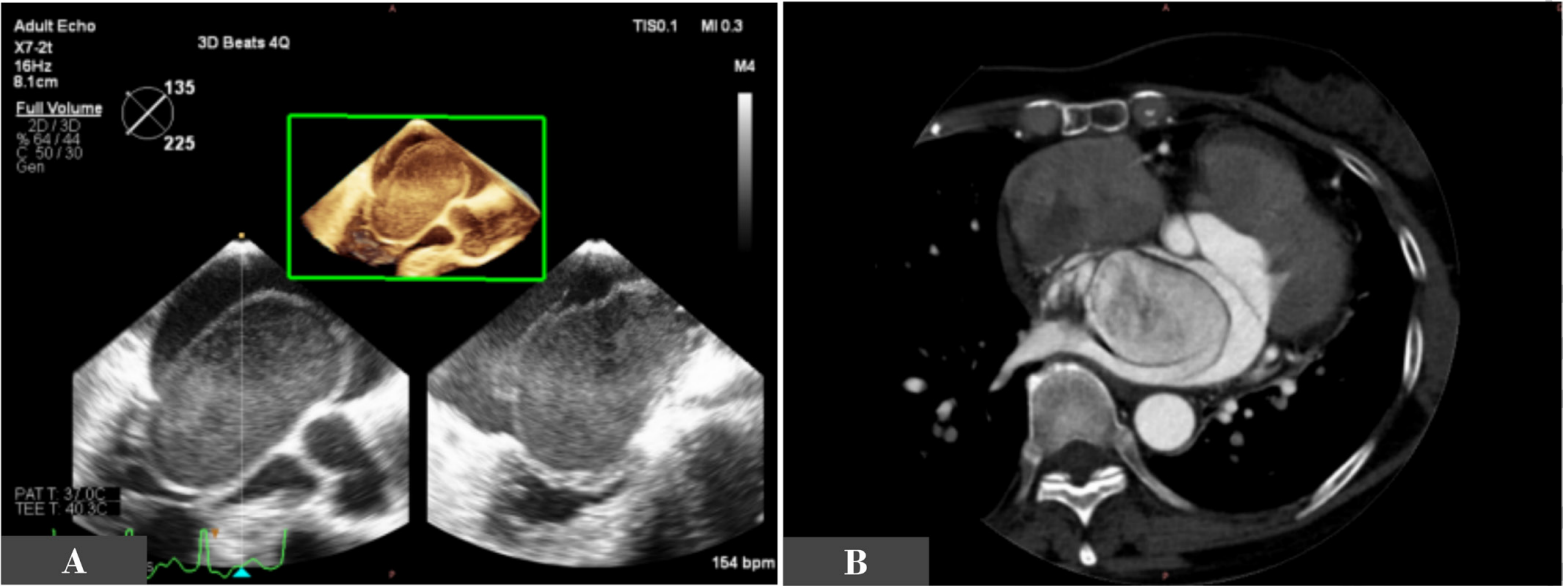
Preoperative three-dimensional transoesophageal echocardiography (A) and cardiac CT in the arterial phase (B).

Observations revealed a normal temperature of 36.5°C and a normal blood pressure of 123/83 mm Hg. A 24-hour 12-lead ECG revealed sinus rhythm, frequent atrial premature beats, paroxysmal atrial flutter with an atrial flutter burden of 9.37% and no paroxysmal ST-T abnormalities. Initial blood tests revealed elevated serum troponin T and B-type natriuretic peptide levels. Transoesophageal echocardiography (TEE) and CT images are shown in figure 1. After the diagnostic procedures, the patient underwent successful surgery.

## Question

What is the most likely diagnosis?

- Cystic formation of the foramen ovale
- Aortic sinus aneurysm
- Left atrial myxoma
- Left atrial appendage aneurysm
- Intracardiac blood cyst

## Answer: C

Preoperative TEE revealed an oval cystic mass in the large left atrium (LA) measuring approximately 76 mm×36 mm, with a base attached to the atrial septum, a clear outline and spontaneous cyclic movement; the mass escaped into the mitral valve orifice during diastole, causing mitral valve obstruction and returned to the LA during systole (figure 1; online supplemental video S1). Contrast-enhanced echocardiography showed that the contrast agent slowly accumulated in the cyst of the mass (online supplemental video S2). Coronary angiography revealed that the left lateral branch of the coronary artery and the right coronary artery provided collateral supply to the mass (online supplemental video S3).

Cardiac CT revealed an isodense mass in the LA that was significantly enhanced. A tortuous vascular structure was observed around the lesion (figure 1; online supplemental video S4).

The patient underwent successful resection of the mass. The mass had a thin but tough bag structure with a few solid structures at the base. Histopathological sections revealed a mucinous stroma with scattered spindle or stellate tumour cells (online supplemental figure S1). These features were consistent with a cardiac mucinous tumour.^1^

Atrial myxomas usually do not have a cystic component.^2^ However, this case was unique, involving a giant cardiac cystic myxoma. Its formation was dependent on three exceptional conditions: blood supply from the feeding arteries into the cyst, adequate size of the drainage and outlet holes, and durability of the cystic membrane. According to the current literature, the proportion of cystic components in this type of myxoma may be the largest among myxomas, which makes preoperative diagnosis difficult.^3^

## supplementary material

10.1136/heartjnl-2024-324714 online supplemental file 1

10.1136/heartjnl-2024-324714 online supplemental file 2

10.1136/heartjnl-2024-324714 online supplemental file 3

10.1136/heartjnl-2024-324714 online supplemental file 4

10.1136/heartjnl-2024-324714 online supplemental file 5
