# Supplementary figures and images for: A rare case of atrial flutter and a cystic mass in the left atrium
Source: Heart. 2024 Sep 25;110(20):e324714. doi: 10.1136/heartjnl-2024-324714 (PMC11503068; doi:10.1136/heartjnl-2024-324714)

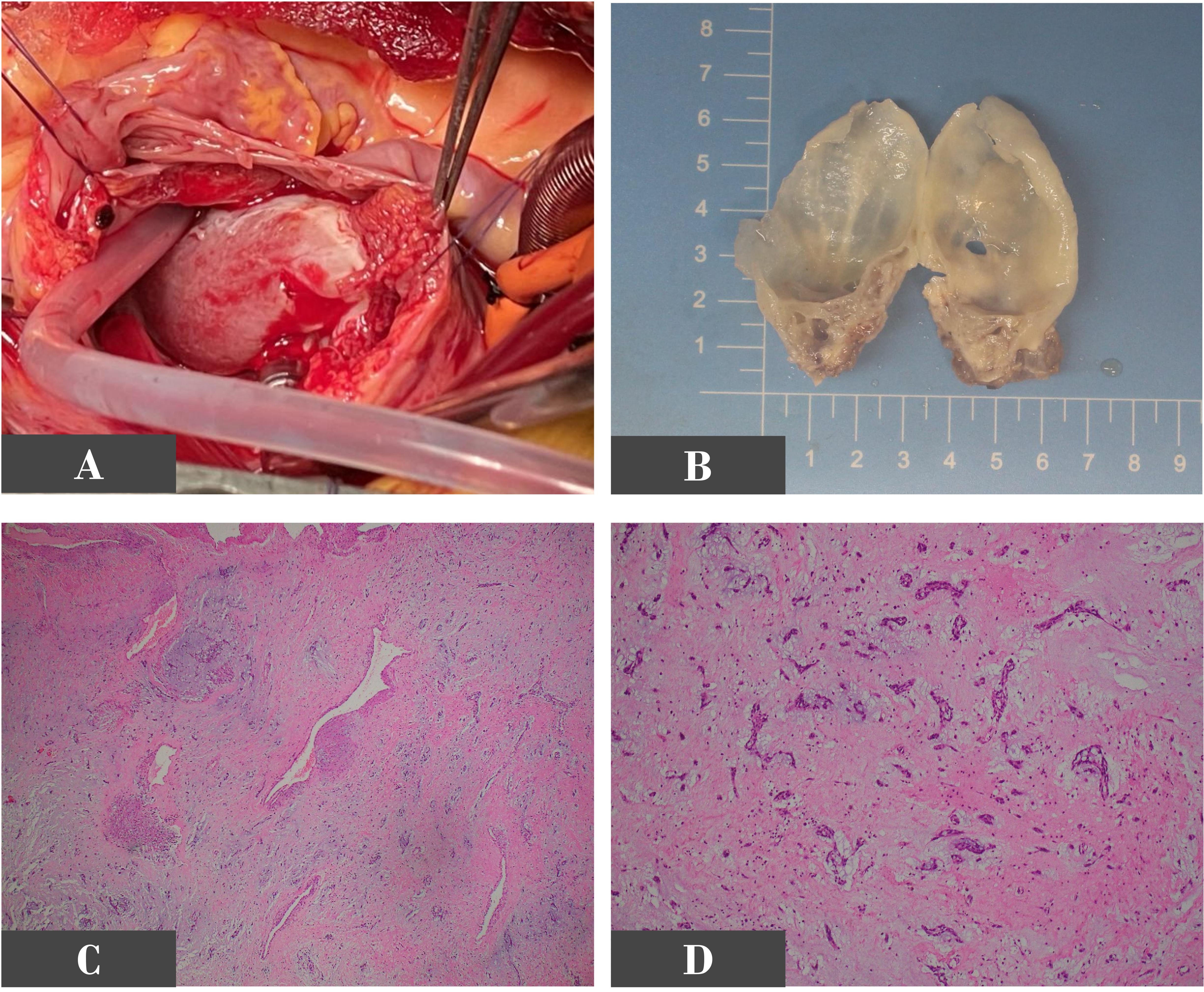

Supplement: online supplemental file 1 [file heartjnl-110-20-s001.jpg]
